# Future trajectory of SARS-CoV-2: Constant spillover back and forth between humans and animals

**DOI:** 10.1016/j.virusres.2023.199075

**Published:** 2023-02-28

**Authors:** Xinhua Cui, Yang Wang, Jingbo Zhai, Mengzhou Xue, Chunfu Zheng, Lu Yu

**Affiliations:** a State Key Laboratory of Human-Animal Zoonotic infectious Diseases, Key Laboratory of Zoonosis Research, Ministry of Education, College of Veterinary Medicine, Center of Infectious Diseases and Pathogen Biology, Department of Infectious Diseases, First Hospital of Jilin University, Changchun, China; b Institute of Animal Health, Guangdong Academy of Agricultural Sciences, Key Laboratory of Livestock Disease Prevention of Guangdong Province, Scientific Observation and Experiment Station of Veterinary Drugs and Diagnostic Techniques of Guangdong Province, Ministry of Agriculture and Rural Affairs, Guangzhou, China; c Medical College, Inner Mongolia Minzu University, Tongliao, China; d Department of Cerebrovascular Diseases, The Second Affiliated Hospital of Zhengzhou University, Zhengzhou, China; e Department of Microbiology, Immunology and Infectious Diseases, University of Calgary, Calgary, Alberta, Canada; f Key Laboratory of Zoonose Prevention and Control at Universities of Inner Mongolia Autonomous Region, Tongliao, China

**Keywords:** SARS-CoV-2, Zoonotic, Transmission, Spillover

## Abstract

SARS-CoV-2, known as severe acute respiratory syndrome coronavirus 2, is causing a massive global public health dilemma. In particular, the outbreak of the Omicron variants of SARS-CoV-2 in several countries has aroused the great attention of the World Health Organization (WHO). As of February 1st, 2023, the WHO had counted 671,016,135 confirmed cases and 6,835,595 deaths worldwide. Despite effective vaccines and drug treatments, there is currently no way to completely and directly eliminate SARS-CoV-2. Moreover, frequent cases of SARS-CoV-2 infection in animals have also been reported. In this review, we suggest that SARS-CoV-2, as a zoonotic virus, may be frequently transmitted between animals and humans in the future, which provides a reference and warning for rational prevention and control of COVID-19.

## Highlights

• SARS-CoV-2 cross-species transmission.
• The constant prevalence of SARS-CoV-2 and its variants.
• The factors SARS-CoV-2 spread.

## 1 Introduction

Severe Acute Respiratory Syndrome Coronavirus-2 (SARS-CoV-2, formerly called HCoV19 or 2019-nCoV), the causative pathogen of Coronavirus Disease-2019 (COVID-19), which was first detected in Wuhan, Hubei Province, China, from December 2019 to March 2020 (Lai et al., 2020). On March 11, 2020, the World Health Organization (WHO) declared COVID-19 a global pandemic (Cucinotta and Vanelli, 2020). Recently, the outbreak of the Omicron variants of SARS-CoV-2 in several countries has affected the global COVID-19 epidemic. Coronaviruses (CoVs) that can infect humans contain HKU1, NL63, OC43, 229E, SARS (severe acute respiratory syndrome)-CoV, and MERS (middle east respiratory syndrome)-CoV, of which SARS-CoV and MERS-CoV are zoonotic and have triggered ultra mortality epidemics during the past 20 years (Low et al., 2021). The two dominant CoV syndromes are clinically known as SARS and MERS. SARS-CoV-2 receptors are primarily expressed in bronchial transient secretory cells and lung tissue, and the virus mainly targets the upper and lower respiratory tracts and spreads to other organs (Lukassen et al., 2020). According to a large cohort study of nine SARS-CoV-2 variants infected 1760 cases in Marseille, France, infection-related deaths were higher in stage 2 (the pulmonary stage) than in stage 1 (the early stage of infection) and stage 3 (the stage of hyper-inflammation) (Hoang et al., 2021). The SARS-CoV-2 transmission was extended by person-to-person linkages of droplets, either directly or through coughs and sneezes. There was a strong correlation between the low per capita mortality rate and the early implementation of mask policies and strict national border control measures (Zeng et al., 2020). However, problems such as the rapid emergence of SARS-CoV-2 variants and cross-species transmission have posed great challenges to animal protection. Some studies suggest that animals worldwide suffer or have already suffered irreparable damage from SARS-CoV-2 (Song et al., 2020; Amy, 2021). However, the exact details of the SARS-CoV-2 transmission between humans and animals remain a mystery. In addition, we aim to discuss whether the SARS-CoV-2 transmission is back and forth between human beings and animals.

## 2 Effects of the SARS-CoV-2 pandemic on humans

### 2.1 A SARS-CoV-2 pandemic in humans

**Fig. 1:**
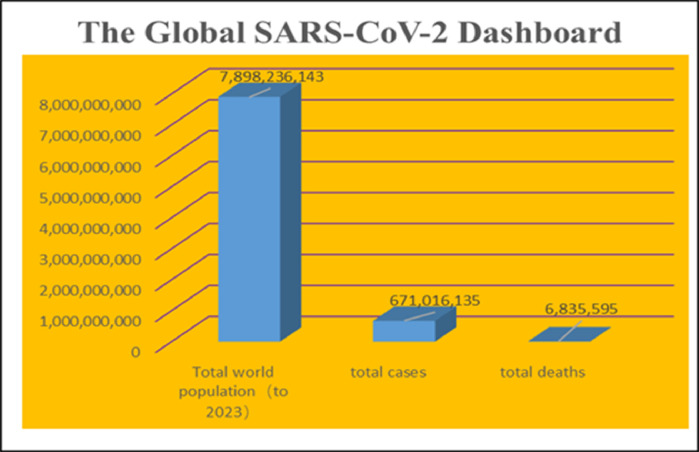
Global SARS-CoV-2 Dashboard. (*https://coronavirus.jhu.edu/map.html*). According to incomplete statistics in 2023, the global population is 7898,236,143 people. As of February 1st, 2023, the WHO had counted 671,016,135 confirmed cases and 6835,595 deaths worldwide.

Currently, the global COVID-19 pandemic caused by SARS-CoV-2 infection is occurring (Haleem et al., 2020). As of February 1st, 2023, the WHO had counted 671,016,135 confirmed cases and 6835,595 deaths worldwide. Fig. 1 shows the global distribution of COVID-19 (CSSE, 2023). In the pandemic's first, second, third, fourth, and fifth waves, people of different races, ages, and occupations were infected. However, there was racial inequality in mortality rates between black and white British groups (Nafilyan et al., 2021). Noteworthily, SARS-CoV-2 mainly attacks young people aged 10–18, pregnant women, and the elderly, perhaps owing to improved diagnostic procedures and tools, longer follow-up, more contagious variants, and different regions (SAL et al., 2021). For example, when COVID-19 began to spread in Tunisia, it was reported that both parents and children were affected (Bourgou et al., 2022). Similar cases have been reported in children in South Korea (Mun and So, 2022). Notably, most of the findings of published research on COVID-19 are in pregnancy and infants (Llorca et al., 2021). Through investigation, most scientists largely agree that pregnancy was developing a specific immunological long-lived memory against SARS-CoV-2 since 14 of the 1167 women recruited tested positive for SARS-CoV-2 RNA on the day of delivery, and subsequently, all 14 children born from these women diagnosed were negative for SARS-CoV-2 RNA (Fenizia et al., 2022). In addition, people with other complications were more vulnerable to SARS-CoV-2 infection, depending on the individual's circumstances. The SARS-CoV-2 infection has been shown to adversely affect dermatologic patients with severe inflammatory disorders or skin cancers and those with prolonged confinement and severe neuropsychiatric symptoms (Numbers and Brodaty, 2021; Wollina, 2020). Typically, there was also evidence of a higher seroprevalence of SARS-CoV-2 among healthcare workers (HCWs) compared to the community at the pandemic's epicenter in a New York City hospital (Venugopal et al., 2021).

### 2.2 New and emerging genetically diverse SARS-CoV-2 Omicron lineages

The new Omicron variants have attracted worldwide attention because of the emergence of many mutations, increasing its transmission and immune evasion ability. As of June 2022, the B.1.1.529 (Omicron) variant of SARS-CoV-2 has been divided into five distinct sublineages: BA.1, BA.2, BA.3, BA.4, and BA.5 (20). The three most typical ones with BA.2.12.1 (hCoV-19/USA/NY-MSHSP-PV56475/2022, first detected in the USA) and BA.4 and BA.5 (hCoV-19/USA/MD/HP30386/2022; hCoV-19/Japan/TY41–702/2022, first detected in South Africa) are currently defeating the previously circulating BA.1 and BA.2 subvariants in several countries, which is putting great pressure on society. These three sublineages exhibit higher transmissibility than the BA.2 lineage (Chen et al., 2021). In particular, the BA.4 and BA.5 of SARS-CoV-2 sublineages were detected as the fifth wave in South Africa, which took place about four months after the Omicron wave (Tegally et al., 2022).

The strong neutralization evasion of BA.4 and BA.5 suggests that immune evasion variants are more likely to be transmitted in populations vaccinated with or recovered from Omicron than BA.4 or BA.2 (Arora et al., 2022). It has been reported that the neutralization of SARS-CoV-2 Omicron BA.4 and BA.5 sublineages was significantly reduced in sera from triple-vaccinated individuals of AstraZeneca or Pfizer vaccine compared with BA.1 and BA.2 (Tuekprakhon et al., 2022). Cao et al. showed that BA.2.12.1, BA.4, and BA.5 exhibit similar binding affinities to BA.2 for the angiotensin-converting enzyme 2 (ACE2) receptor (Cao et al., 2022). In addition to the known mutations in BA.2, the spike proteins of BA.2.12.1 also contain L452Q and S704L mutations, while the spike proteins of BA.4 and BA.5 are the same, and each has four more alterations: Del69-70, L452R, F486V and R493Q, which is a reverse mutation (Wang et al., 2022). However, most of these cross-reactive neutralizing antibodies are evaded by spike mutants L452Q, L452R, and F486V (Wang et al., 2022). Substitutions L452Q, L452R, and F486V are in the receptor-binding domain of the spike protein, the major target for monoclonal antibody therapies, which is problematic concerning the effectiveness of current monoclonal antibodies that have been approved by the Food and Drug Administration (FDA) against these variants. Furthermore, mutation at spike residue L452 found in both BA.2.12.1 and BA.4/5 facilitates escape from some antibodies directed to the so-called class 2 and 3 regions of the receptor-binding domain (Christopher et al., 2020). Neutralizing antibody titers against the BA.4 or BA.5 subvariant and (to a lesser extent) against the BA.2.12.1 subvariant was lower than titers against the BA.1 and BA.2 subvariants, which suggests that the SARS-CoV-2 omicron variant has continued to evolve with increasing neutralization escape (Hachmann et al., 2022). Through neutralization sensitivity analysis of *pseudovirus*, Wang *et al*. showed 21 monoclonal antibodies (mAbs) targeting known neutralizing epitopes on the SARS-CoV-2 viral spike, 18 and 19 mAbs completely or partially lost their neutralizing activity against BA.2.12.1 and BA.4/5, respectively (Wang et al., 2022).

Even so, the three-injection vaccine can still effectively prevent Omicron to some extent, although a certain proportion of breakthrough infections have mild symptoms. Some countries have approved implementing a fourth injection vaccine, which can restore the attenuated protective effect of the third dose of vaccine over time (Regev et al., 2022). Fortunately, the three small-molecule antiviral drugs remdesivir, molnupiravir, and nirmatrelvir may have therapeutic value against the sublineages BA.2.12.1, BA.4, and BA.5 of SARS-CoV-2 Omicron variants. However, it still requires extensive clinical trials (Takashita et al., 2022). Fan *et al*. believe that developing new neutralizing antibodies against relatively conserved sites is still an effective choice to deal with the emerging variants of SARS-CoV-2 (such as Omicron) (Fan et al., 2022). Homologous or heterologous boosters and new vaccines against Omicron, as well as possible novel variants, have been proposed to improve protection against vaccine failure. In addition, social distance restriction is still an effective strategy to prevent and control the spread of the Omicron variant (Fan et al., 2022).

## 3 Effects of SARS-CoV-2 on animals

### 3.1 A SARS-CoV-2 pandemic in animals

SARS-CoVs are high-impact respiratory pathogens of animal origin that can also make animals sick (Sooksawasdi et al., 2021). Richard et al. tested RNA from 418 bats, and 17% (71/418) of the bats were positive for viral RNA from one or more viruses, including *paramyxoviruses, coronaviruses*, and other unknown zoonotic potential viruses (Suu et al., 2022). A pandemic caused by the adult *equine coronavirus* (ECoV) CH21 strain has occurred in Switzerland (Barroso et al., 2022). Still, Alkhovsky *et al*. reported that a SARS-like coronavirus has spread among horseshoe bats in Russia (Alkhovsky et al., 2022). Recent SARS-CoV-2 susceptibility studies in cats in the United States have shown that the virus can replicate in companion animals and spread to other cats (Natasha et al., 2020). The researchers found that cattle, buffalo, goats, and sheep, the dominant species in south Asian family farming systems, were potentially at risk of infection with SARS-CoV-2. However, all birds studied with rats and mice were considered less likely to interact with SARS-CoV-2 (Ahmed et al., 2021). Smith et al.found that mice infected with the SARS-CoV-2 B1.351 variant showed symptoms ranging from mild to fatal, depending on their age (Yasui et al., 2022).

Viruses exposed in high-density populations can spread continuously among susceptible animal species. For example, adult dairy goats have reported outbreaks of diarrhea associated with coronavirus infection (F L Smith et al., 2022). Guilherme et al. have provided evidence that SARS-CoV-2 particles in freshwater ecosystems pose health risks to fish and other aquatic organisms (Malafaia et al., 2022). In addition, antibodies to COVID-19 IgG were detected in 35/587 (5.9%) thoroughbred racing horses from California collected between July 10 and September 12, 2020 (Lawton et al., 2022). Although this study showed low SARS-Cov-2 antibody titers in horses, it may be related to potential host variability and host acceptance. Therefore, it is important to investigate further the possibility of SARS-CoV-2 and its variants being infected in other animals.

### 3.2 Effects of SARS-CoV-2 on animals

SARS-CoV-2 also has fatal effects on animals. In developing countries, the COVID-19 crisis has severely impacted poultry production and sustainability (Attia et al., 2022). As a result, Pakistan has raised the need for national livestock surveillance for COVID-19 (Aamir Shahzad, 2022). Some scholars suggested that it is also necessary to strengthen the control of mink farms to prevent the emergence of new SARS-CoV-2 variants in this animal reservoir (Colson et al., 2021). It is particularly worrying that despite the global SARS-CoV-2 pandemic, consumer demand for exotic pets has not decreased, so global demand for wildlife needs to be curtailed (J Ribeiro et al., 2022). Ccompanion animals also require veterinary care during the global pandemic SARS-CoV-2 (Muzzatti and Grieve, 2022). Some data suggest that SARS-CoV-2 increases susceptibility and pathogenicity to bacterial co-infection (A P Smith et al., 2022). However, *non-typhoidal Salmonella, Staphylococcus aureus*, and *enteropathogenic Escherichia coli* decreased somewhat, but not significantly, compared with the previous two years (Ahn et al., 2021). Risk factor identification and serological monitoring of the group-housed animal species susceptible to SARS-CoV-2 may help reduce the threat of reverse zoonosis caused by COVID-19.

## 4 Cross-species transmission of SARS-CoV-2

### 4.1 Intermediate host of SARS-CoV-2

A wide variety of animal species are sensitive to SARS-CoV-2, suggesting that the virus can cross the species barrier and adapt to survive. Silicon infection analysis (ISFA) system was used to analyze the binding affinity and interaction interface between the S1 protein and ACE2 of different species, and it was found that SARS-CoV-2 had inter-species and cross-species transmission potential (Zou et al., 2022). Therefore, it is essential to identify how potential intermediate hosts participate in the viral transmission chain. Given indirect evidence for the possibility of an initial zoonosis emergence, it is too early to determine the role of intermediate hosts such as snakes, pangolins, turtles, and other wild animals in the origin of SARS-CoV-2, excluding bats, which are the natural hosts of multiple coronaviruses such as MERS-CoV (Ruchi et al., 2020). Royce *et al*. proposed that mink, pangolin, and ferret are the intermediate hosts of SARS-CoV-2 through a new mathematical model method (Royce, 2021). However, molecular biology and phylogenetic analysis showed that pangolin coronavirus (pangolin-CoV-2020) was genetically related to SARS-CoV-2, but SARS-CoV-2 was not directly derived from pangolin-CoV-2020 (P Liu et al., 2020; Xiao et al., 2021). The researchers used a double-antigen sandwich ELISA to detect SARS-CoV-2 specific antibodies in 1914 sera from domestic livestock (pig, cow, sheep, horse), poultry (chicken, duck, goose), experimental animals (mice, rat, guinea pig, rabbit and monkey), companion animal (dog and cat) and wild animals (camel, fox, mink, alpaca, ferret, bamboo rat, peacock, eagle, tiger rhinoceros, pangolin, leopard cat, jackal, giant panda, masked civet, porcupine, bear, yellow-throated marten, weasel, red pandas and wild boar) were excluded as intermediate hosts of the SARS-CoV-2 (Deng et al., 2020).

### 4.2 Cross-infection of SARS-CoV-2 between humans and animals

#### 4.2.1 Animal-to-human transmission of SARS-CoV-2

SARS-CoV-2 is thought to be able to spread between animals and humans by crossing the species barrier, causing a pandemic (Mahdy et al., 2020). Several studies have shown that exotic animals with a high number of species can spread the coronavirus to humans, especially in areas where exotic animals are used for food (J Ribeiro et al., 2022; Rotstein et al., 2022; Shankar et al., 2021). Animals sold for human food consumption are the first source of transmission of the current COVID-19 outbreak. The first patients in the COVID-19 outbreak in Wuhan, Hubei Province, China, were workers or buyers of the city's main animal market who had previously had extensive contact with animals, suggesting that SARS-CoV-2 could be transmitted from animals to humans (Lin et al., 2020).

Nkom *et al*. found that Cameroon carries multiple coronaviruses, including many closely related to human coronavirus 229E (Ntumvi et al., 2022). Munnink *et al*. reported that 68% of mink farm residents, employees, and/or individuals who had contact with them were found to have evidence of SARS-CoV-2 infection through whole-genome sequencing (BB Oude Munnink et al., 2021). In addition, Chan *et al*. reported that the SARS-CoV-2 Delta variant AY.127 may have been transmitted from animals to humans, resulting in a COVID-19 outbreak in a pet shop in Hong Kong (Jasper et al., 2022). Through multidisciplinary research cooperation on SARS-CoV-2 monitoring in wild animals in Canada, it was found that there were high differences in SARS-CoV-2 lineage, which have 76 consensus mutations (including 37 previously associated with non-human animal hosts) and a large number of features that have evolved and spread in wildlife. This survey first demonstrated the continued evolution of SARS-CoV-2 in white-tailed deer and deer-to-human transmission (Bradley et al., 2022). Because the SARS-CoV-2 pandemic has not been effectively controlled, investigating and controlling the transmission of SARS-CoV-2 from animals to humans is of great significance.

#### 4.2.2 Human-to-animal transmission of SARS-CoV-2

**Fig. 2:**
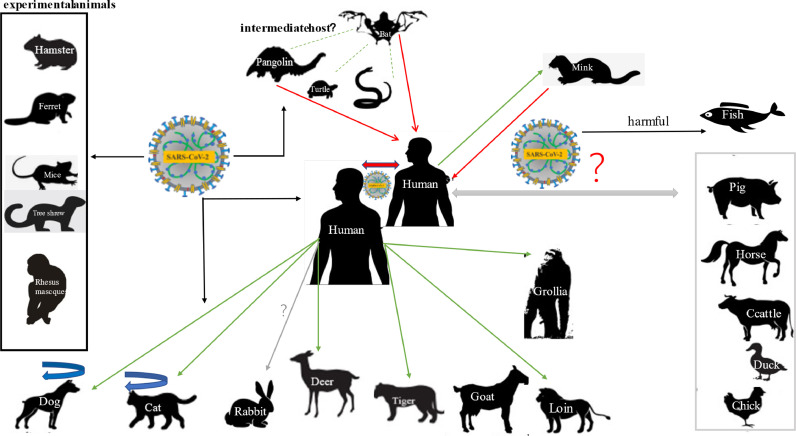
The circulation of SARS-CoV-2 in human beings and animals. The double red arrows represent transmission human-to-human, the thick blue arrows represent transmission from species to species, the green arrows represent transmission animal-to-human, the red arrows indicate transmission animal-to-human, the SARS-CoV-2, and the black arrows represent its variants, and the gray arrows are unknown.

Humans remain the most likely source of SARS-CoV-2 transmission to other humans and domestic, zoo, and wild animals. Human-to-animal transmission has been reported by cats, dogs, tigers, lions, minks, and deer. A report has revealed that a tiger and a lion at the Bronx Zoo in New York, the United States, were infected with SARS-CoV-2 transmitted by a breeder (McAloose et al., 2020). Similarly, scholars found that in several mink farms, SARS-CoV-2 jumped back and forth between humans and minks (Lu et al., 2021; BB Oude Munnink et al., 2021). Companion animals, especially cats and dogs, can be infected with SARS-CoV-2 (Jian et al., 2020), and whole-genome sequencing revealed that SARS-CoV-2 was identical in dogs and humans, suggesting human-to-animal transmission (Decaro et al., 2021). In addition, SARS-CoV-2 was detected in Asiatic lions from a zoo in India, and sequence and phylogenetic analysis showed that the virus was a B.1.617.2 (Delta) variant (Mishra et al., 2021). The pathogenicity of B.1.617.2 (Delta), B.1.617.3 lineage of SARS-CoV-2, and the early B.1 virus carrying the D614G mutation were compared in a Syrian hamster model, although there was no significant difference in the shedding pattern of viruses across variants, high levels of SARS-CoV-2 subgenomic RNA were detected in the respiratory tract of hamsters infected with the Delta variant for 14 days, suggesting that further transmission studies are needed (Mohandas et al., 2021). Based on 3.2.1 and 3.2.2, we illustrate the possible transmission process of SARS-CoV-2 in humans and animals in Fig. 2.

### 4.3 The secret of SARS-CoV-2 cross-species transmission

#### 4.3.1 Evolution and recombination of SARS-CoV-2

Data from early outbreaks and hospital records are needed to track the evolutionary path of SARS-CoV-2 and identify the key steps needed for its effective transmission. Until now, the origin of SARS-CoV-2 can be unequivocally traced to horseshoe bats, genus *Rhinolophus* (Lytras et al., 2022). Through phylogeny, metagenomic data, and other methods, SARS-CoV-2 belongs to the Coronaviridae family (Galkin et al., 2022). Yang *et al*. noted that pangolin SARS-CoV-2-related viruses were divided into two sub-lineages based on geographic sampling sites (Yang et al., 2021). Furthermore, the SARS-CoV-2 virus belongs to the *Betacoronaviruses* genus, and phylogenetic evidence showed that SARS-CoV-2 could develop from bat-SL-CoVZC21 (Lohrasbi, 2022). Biazzo *et al*. obtained the genome sequences of 10 SARS-CoV-2 virus strains through nanopore sequencing of nasopharyngeal swabs in Malta and analyzed the assembled genome with pangolin software, and the results showed that these virus strains were assigned to B.1 lineage, indicating that SARS-CoV-2 was widely spread in Europe (Biazzo et al., 2021). Cilibrasi *et al*. analyzed the genome-wide phylogeny and classification of SARS-CoV-2 using the normalized compression distance (NCD) method, and the results showed that SARS-CoV-2 was 96.2% homologous to the RaTG13 virus and similar to two bat SARS-like coronaviruses bat-SL-CoVZXC21 and bat-SL-CoVZC4 (Cilibrasi and Vitanyi, 2020). Lopez *et al*. demonstrate that SARS-related coronaviruses, such as SARS-CoV-2, are distributed over large geographical areas in southern China and Southeast Asia. During their evolutionary history, they have undergone a great deal of recombination, showing frequent transmission among their *Rhinolophus* host species (Lopez et al., 2021). Tegally *et al*. speculated that with the continuous discovery of the Omicron lineage of SARS-CoV-2 with genetic diversity, the support level of the hypothesis about its origin has changed, and it has been transferred from an unselected location to a discrete reservoir, such as human chronic infection (or even chronic human infection network) and/or animal reservoir, which may contribute to the further evolution and spread of the SARS-CoV-2 (Tegally et al., 2022). In addition, Khadka *et al*. demonstrated that the conserved molecular characteristics of S protein provided evidence for the origin of SARS-CoV-2 and Pangolin-CoV (MP789), produced by recombination between specific SARS virus lineages (Kreuder Johnson et al., 2015).

**Fig. 3:**
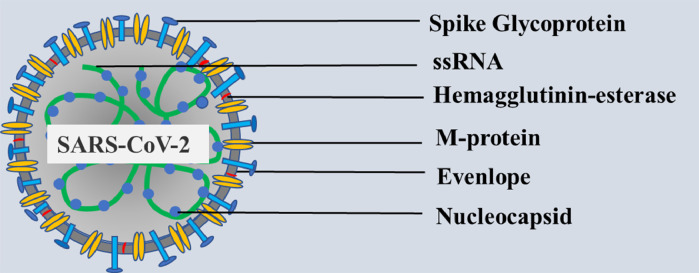
The structure of SARS-CoV-2. As shown in the figure, the structure of SARS-CoV-2 concludes spike glycoprotein, envelope, membrane, and nucleocapsid.

Goh *et al*. believe that SARS-CoV-1/2 may have been secretly transmitted in the human body for many years before it was discovered and evolved along the zoonotic pathway to human infection of SARS-CoV-1/2. The qualitative timeline shows that the ancestral strain of SARS-CoV-1/2 needs a long time before it evolves into transmission between pangolins and humans (Lopez et al., 2021). Once the virus spreads between humans and animals, it will cause considerable social and ecological pressure. Eleven SARS-CoV-2 genomes have been sequenced successfully, and all genomes carried the spike mutation D614G and were classified as part of the GH clade in Congo (Ntoumi et al., 2021). Pangolin macrogenome data showed the genetic relationship with SARS-CoV-2, *Sendai virus, flavivirus, picornavirus, parvovirus*, and *genomovirus*, and these viruses' genomes exhibited the signal of genome recombination (Goh et al., 2022). It was shown that SARS-related coronavirus evolution is mainly driven by the acquisition and selection of spike substitutes with biological advantages, social constraints, and population size, which is a key factor in explaining the epidemic pattern in the Balearic Islands (Lopez et al., 2021). Fig. 3 describes the structure of SARS-CoV-2.

#### 4.3.2 Interaction between ACE2 and spike (S) glycoprotein

Zhai *et al*. showed that if the ACE2 of SARS-CoV-2-infected species is tolerant of various amino acid changes, it indicates that the species barrier is low and the risk of SARS-CoV-2 cross-species transmission is high (Zhai et al., 2020). Nearly all mammal species susceptible to SARS-CoV-2 infection (cats and ferrets) have many amino acid mutations in their ACE2 proteins. Noteworthily, even if there are a large number of amino acid changes (such as 5 amino acid changes found in pig ACE2) on the interaction surface of SARS-CoV-2 S protein-protein, it will not affect the binding of ACE2 in some animals (Zhai et al., 2020). Some species, especially those closely related to humans, are at risk of infection with SARS-CoV-2, which may establish additional virus banks in one of the various animals. ACE2 of humans, pangolin, cats, and bats interact with the receptor-binding domain (RBD) S protein of SARS-CoV-2 (Ma et al., 2021). The evolution of SARS-CoV-2 requires tight RBD-ACE2 binding and effective RBD confirmation sampling to obtain deep infection (Zhang et al., 2021). Piplani et al. carried out in silico structure homology modeling, protein-protein docking, and molecular dynamics simulation of the ability of SARS-CoV-2 S protein to bind ACE2 and found that relatively high species (monkeys, hamsters, dogs, ferrets, and cats) have an implicit effect on SARS-CoV-2 infection, supporting the association between binding affinity and infection susceptibility (Piplani et al., 2021).

Kyle et al. described the SARS-CoV-2 spike protein binding to its protein receptor ACE2 through a review and how the host protease primes and then makes the effective membrane fusion between the virus and the target cell (Kyle et al., 2022). A cryo-EM (cryO-EM) result showed that the conformational switch of a domain of CTD1, the S protein subunit, was a prerequisite for receptor binding (Song et al., 2018). The binding of the receptor further opens up CTD1 to expose other structures. The Spike fragment of SARS-CoV-2 peptides can cause genomic instability and erythrocyte DNA damage (Goncalves et al., 2022). Furthermore, in the genomic characteristics and phylogenetic analysis of the first batch of SARS-CoV-2 variants introduced in Lebanon, single nucleotide variants (SNVs) analysis showed 15 new mutations, of which only one was detected in the S protein region (Feghali et al., 2021). Vilcek *et al*. clarified that the analysis of the SARS-CoV-2 genome, especially the S gene, showed that the natural evolution process between bat-CoV and a pangolin-CoV or other animal coronaviruses might be of great significance in creating SARS-CoV-2 and transmitting new viruses to human populations (Vilcek, 2020). Paniz *et al*. showed by genome sequencing that the SARS-CoV-2 lineages from Venezuela are similar to the virus collected from patients in the border areas of Colombia and Brazil, consistent with the cross-border transit despite administrative measures, including a blockade. They also reported mutations associated with increased infectivity in three Venezuelan genomes and the Colombian SARS-CoV-2 genome from adjacent border areas (Paniz et al., 2020). Therefore, it may be possible to predict the new mutation sites in the future by analyzing the close relatives (outgroups) of SARS-CoV-2 infected with non-human hosts (Katoh and Standley, 2021).

#### 4.3.3 Spillover theory

With the spread of the SARS-CoV-2 pandemic, a question has been raised: are infectious diseases related to environmental changes? Researchers found that livestock and humans share many viruses. That is, viruses have high host plasticity and adaptation (Khadka and Gupta, 2021). Coronavirus in wild and domestic animals has a long history of spillover among species (Cui et al., 2019), which may result from frequent human interaction with these animals for centuries.

On the one hand, with the increase in population, wild animals that can adapt well to the human-dominated environment will also share more viruses with humans, including some rodents, bats, and primates (Decaro and Lorusso, 2020). The population explosion has expanded the scope of human activities and occupied most of the living space previously reserved for animals, which leads to frequent contact between humans and animals. They live around human houses, farms, and crops. They are high-risk species of spreading the virus to humans. On the one hand, with the increase in population, wild animals that can adapt well to the human-dominated environment will also share more viruses with humans, including some rodents, bats, and primates. The population explosion has expanded the scope of human activities and occupied most of the living space previously reserved for animals, which leads to frequent contact between humans and animals (Decaro et al., 2020). These animals live around human houses, farms, and crops and are high-risk species for spreading the virus to humans.

On the other hand, some reasons are related to hunting, wildlife trade, and habitat quality decline. According to the canyon hypothesis of zoonotic spillover, in continuous transmission between seronegative individuals, the large virus population size and extensive transmission bottleneck contribute to the rapid emergence of variants conducive to the rapid transmission of open RBD structure between hosts (Rahimi et al., 2021). It is predicted that these species carry twice the number of zoonotic viruses as endangered species for other reasons.

## 5 Will SARS-CoV-2 exist for a long time?

COVID-19 outbreak shows that animal-to-human contact may be the main source of emerging zoonotic infectious diseases, and its specific risk factors include sufficient interaction between infected people and recipient animals, the suitability of animal host factors for productive virus infection, and the suitability of animal host population for virus persistence (Alex et al., 2021; Zhai et al., 2022). In particular, the main problem is that the virus spreads to susceptible animal species. In these species, group residence and contact network structure may lead to another virus library, which can be reintroduced into humans (Aleta et al., 2020). At least 10,000 viruses in the world can infect humans, but most of them used to spread quietly among wild mammals, and climate change will promote the sharing of new viruses among mammalian species (Colin et al., 2022). However, we should determine the exact mechanism of zoonotic diseases and their initial transmission, which will help us design and implement appropriate prevention barriers to prevent the further transmission of SARS-CoV-2 (Tiwari et al., 2020). We all know that the reproduction number (R0) means viral transmissibility. R0 > 1 indicates that an infected person has produced many new infections, and the epidemic will increase; If R0 < 1, the transmission will disappear. WHO estimated that the reproduction number of COVID-19 ranged from 1.4 to 2.5, although a new study found that the average R0 of COVID-19 is 3.2823 (Y Liu et al., 2020). Thus, SARS-CoV-2 does not seem to disappear from the earth soon and may spread back and forth frequently between humans and animals.

## 6 Future perspectives

For the role of other animals in the COVID-19 pandemic, especially cattle, sheep, goats, horses, and donkeys, there is a knowledge gap, which should be determined through targeted monitoring. Technological advances such as the detection and treatment of COVID-19 are constantly innovating to make its future epidemic easier to control. The vaccines, antibody therapies, and antiviral drugs approved so far are based on decades of investment in technology and basic science. There are dozens of ongoing preclinical and clinical trials, the main purpose of which is to reuse approved drugs and potential candidate compounds to target SARS-CoV-2 host factors and their current status (Tharappel et al., 2020). In addition, vaccination is a way to prevent virus infection, but it still needs further clinical verification. Even with vaccinations, the virus may reinfect (Raveendran, 2021). Moreover, vaccinated individuals reinfected with SARS-CoV-2 may be the source of transmission (Schiavone et al., 2021).

Although vaccination of high-risk populations is considered an instrumental model to block the pandemic, non-drug interventions, including avoiding mass gatherings, school closures, case isolation, contact tracing, and infection prevention strategies in a healthcare setting, are the cornerstone of preventing transmission (Mouliou and Gourgoulianis, 2021). Combining lessons from previous pandemics, the public health response to the COVID-19 pandemic is the basis for best practices against future pandemics (Mujica et al., 2020). On the premise of protecting wild animals, minimizing human contact with wild animals is also important to reduce the risk of coronavirus transmission from wild animals to humans. We should be well prepared for the future pandemic and minimize their impact. We should be fully prepared for the future COVID-19 pandemic and minimize its impact. To sum up, to live in harmony between humans and animals, we still need to explore the secret of the SARS-CoV-2 virus.

## Summary

Our review describes the cross-species transmission of SARS-CoV-2 and its impact on humans and animals. We summarized the epidemic situation of SARS-CoV-2 and its variants in humans and animals and clarified SARS-CoV-2 constant spillover back and forth between humans and animals with the evolution and recombination, the interaction between ACE2 and Spike (S) glycoproteins, and the spillover theory. We hope these contents can provide coping strategies for SARS-CoV-2, which spreads back and forth between humans and animals.

## Authors' contributions

Xinhua Cui: wrote this article; YangWang, Jingbo Zhai, Mengzhou Xue, Chunfu Zheng, and Lu Yu: Revise and review this article.

## Ethics approval and consent to participate

Not applicable.

## Consent for publication

All authors have given their consent to the publication of this article.

## Availability of data and materials

Not applicable.

## Funding

This work was supported by The 10.13039/501100012166National Key Research and Development Program of China (2021YFC2600200), the Science and Technology Research Project of the Jilin Provincial Department of Education (JKH20211179KJ; 2016444), and the Jilin Provincial Nature Science Foundation of Jilin Provincial Department of Science and Technology (20210101341JC). This work was also supported by the National Natural Science Foundation of China (No. 81801972).

## Declaration of Competing Interest

The authors declare that they have no known competing financial interests or personal relationships that could have appeared to influence the work reported in this paper.

## Data Availability

No data was used for the research described in the article.
